# Biocatalysis on the surface of *Escherichia coli*: melanin pigmentation of the cell exterior

**DOI:** 10.1038/srep36117

**Published:** 2016-10-26

**Authors:** Martin Gustavsson, David Hörnström, Susanna Lundh, Jaroslav Belotserkovsky, Gen Larsson

**Affiliations:** 1 Division of Industrial Biotechnology, School of Biotechnology, KTH Royal Institute of Technology, Albanova University Center, SE 10691 Stockholm, Sweden

## Abstract

Today, it is considered state-of-the-art to engineer living organisms for various biotechnology applications. Even though this has led to numerous scientific breakthroughs, the enclosed interior of bacterial cells still restricts interactions with enzymes, pathways and products due to the mass-transfer barrier formed by the cell envelope. To promote accessibility, we propose engineering of biocatalytic reactions and subsequent product deposition directly on the bacterial surface. As a proof-of-concept, we used the AIDA autotransporter vehicle for *Escherichia coli* surface expression of tyrosinase and fully oxidized externally added tyrosine to the biopolymer melanin. This resulted in a color change and creation of a black cell exterior. The capture of ninety percent of a pharmaceutical wastewater pollutant followed by regeneration of the cell bound melanin matrix through a simple pH change, shows the superior function and facilitated processing provided by the surface methodology. The broad adsorption spectrum of melanin could also allow removal of other micropollutants.

Growing environmental concern requires the design of sustainable processes to replace products that are currently derived from fossil sources and has also promoted the development of new bioremediation methods to handle the continued pollution of air, soil and water. Microorganisms are valuable tools for solving many of these problems, particularly if processes can become economically sustainable. Continued research advances allow the directed evolution of both cells and enzymes and a successful example of transplantation of a complete heterologous pathway is the introduction of *Saccharomyces* and *Klebsiella* enzymes into *Escherichia coli* for the production of 1,3-propanediol^1^.

However, sustainable production requires not only the engineering of new production pathways but also the generation of cells with new characteristic features. Often, such changes are required for low cost cellular growth on waste biomass, such as lignocellulose, that is inaccessible to most production organisms. In an elegant example, the surface expression of enzymes from *Trichoderma* and *Aspergillus* was used to mimic a bacterial cellulosome, providing the required hydrolytic properties needed for ethanol production directly from cellulose in *S. cerevisiae*^2^.

The surface expression of active enzymes in production organisms that are not known exporters, such as *E. coli* laboratory strains, is a challenge. Several translocation systems targeting the cell exterior have been suggested over the years, but very few result in complete excretion in which the protein actually faces the environment^3^,^4^. A vehicle that today has gained much attention is the autotransporter (AT) family, which is an example of type V excretion and used by pathogenic Gram-negative bacteria to excrete virulence and adhesion factors^5^. Dating from discovery in 1987^6^ the AT family has been continuously expanding, making it the most abundant protein translocator class in bacteria^7^. ATs offer an elegant solution to the biggest obstacle for Gram-negative surface display, namely translocation through the outer membrane (OM) in the absence of adenosine triphosphate and without assistance of a proton motive force. This obstacle is overcome by the expression of a single polypeptide bearing all the required functionalities: an N-terminal signal peptide directing the protein to the periplasm, a “passenger” domain responsible for the functionality of each individual AT, and a C-terminal β-barrel that acts as an OM anchor and as a pore for passenger domain transport^7^.

The positioning of enzymes on the surface by use of appropriate mechanisms for expression and translocation to the surface constitutes a new cell modification strategy, highlighting an example of process facilitation by providing external access to the reactant. In addition, the combination of production and excretion reduces the amount of downstream unit operations, in particular enzyme purification and immobilization making the process faster, simpler and cheaper. Even if this strategy permits the introduction of new cellular characteristics, the expression of a single protein will not provide a reasonably large toolbox for the range of modifications that will eventually be required, particularly if the resulting molecules are restricted to proteins. We therefore suggest extending this perspective by running complete biocatalytic reaction pathways on the cell surface that will permit deposition and attachment of a larger variety of end products.

For this purpose we selected the polymerization of melanin. Melanin is a biopolymer produced from the substrate tyrosine through a series of reactions catalyzed by tyrosinase in an oxidative environment^8^. Melanin production occurs naturally in many living cells but not in *E. coli*. The principal purpose of melanin is detoxification, in particular with respect to UV damage^9^. Today, it is well established that pharmaceutical substances pass largely unharmed through conventionally equipped municipal wastewater treatment plants and accumulate in the aquatic ecosystem^10^. Several of these drugs have a proven record of impairing the reproducibility of living species, with fish and frogs as frequently used models^11^. The accumulation of drugs seems to be related to adsorption into lipids or melanin-rich tissues, in particular in the hair and the eyes^9^,^12^. It is since long understood that melanin has affinity to a multitude of pharmaceutical compounds and metal cations^9^,^12^.

This study aimed to create biological adsorptive agents, which utilizes the surface expression methodology for surface biocatalysts and subsequent deposition of melanin. The specific research challenge was threefold: (1) design a vector for the expression and transport of tyrosinase in an active configuration to the outer part of the OM, with proven enzyme exposure to the environment, (2) provide the conditions necessary to catalyze the polymerization reaction and complete oxidation to the polymer by use of this enzyme and (3) use the inherent characteristics of melanin such as its well-known adsorption of pharmaceuticals^9^,^12^ to demonstrate functionality of melanin surface deposition by an appropriate example.

## Results and Discussion

Tyrosinases are a large group of enzymes with multiple characteristics and are generally not qualified or even suitable for surface expression since they are often large multidomain complexes. Furthermore, it is critical to avoid premature folding in the cytosol and periplasm, since neither the inner nor the OM translocation apparatus is large enough to accommodate any degree of secondary structure. The two tyrosinases selected and subsequently investigated were; one with high similarity to the *Bacillus megaterium* native tyrosinase^13^ (Tyr1) (Supplementary text 1) and one identical to the *Rhizobium etli* tyrosinase^14^ (Tyr2). The selection was based on the available structural characterization of the native enzymes and their previous cellular expression albeit in the *E. coli* cytosol^13^,^14^, where membrane transport has been suggested as a limitation for melanin production^15^. Tyr1 was chosen since its relatively small (34 kDa) and lacks cysteines, both known advantages for avoiding premature folding during surface display^16^,^17^,^18^. Tyr2 is larger (67 kDa) and contains 5 cysteines. Finally, in contrast to many other tyrosinases, neither of the chosen enzymes requires auxiliary “caddy” proteins to generate their copper-containing active forms^13^,^14^.

**Figure 1:**
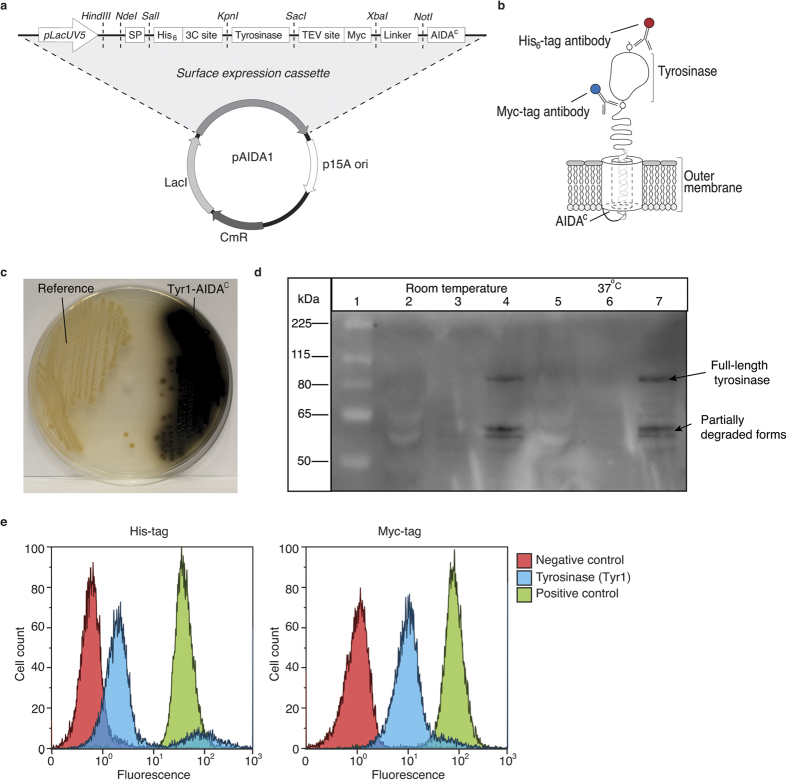
Mechanism for tyrosinase display on the surface of *E. coli*. (**a**) Schematic presentation of the surface expression cassette pAIDA1^17^ based on the AIDA autotransporter. Targeted tyrosinases were inserted into the passenger site using the KpnI and SacI restriction sites. (**b**) Schematic representation of the predicted enzyme position on the cell surface with the flanking peptide tags: His_6_ (six-histidine peptide) and Myc (peptide from the *c-myc* gene) subjected to specific antibody binding. (**c**) Agar plate showing *E. coli* expression of the Tyr1-AIDA^c^ fusion protein compared to a reference (cells with AIDA^c^ lacking passenger). The melanin production is indicated by the black color. (**d**) Cellular protein fractions assayed by Western blotting for the presence of the Myc tag located between Tyr1 and AIDA^c^. The soluble fractions are lanes 2 and 5, the inner membrane protein fractions are lanes 3 and 6 and the outer membrane protein fractions are lanes 4 and 7. (**e**) Probing whole cells expressing Tyr1-AIDA^c^ with fluorescent antibodies against the two epitope tags His_6_ and Myc followed by flow cytometry analysis as compared to the negative control (cells without the surface display vector). The positive control are cells containing the AIDA^c^ expression system without a passenger.

ATs are not an entirely uniform family and for the present purpose we chose the specific autotransporter Adhesin Involved in Diffuse Adherence (AIDA-I) since it has a modest but successful track record for translocation of enzymes^16^,^19^,^20^ and vaccine epitopes^21^. The pAIDA1 vector^22^ (Fig. 1a), including the wild type signal peptide and β-barrel (AIDA^c^), was engineered to facilitate the identification of correct surface display by introducing two reporter sequences (His_6_ and Myc tags) flanking the passenger (Fig. 1b). Furthermore the vector contains a low copy number origin of replication and a weak promotor (lacUV5) to prevent rapid build up of protein intermediates in the cytoplasm prior translocation. The lacUV5 promotor is independent of cyclic adenosine monophosphate mediated catabolite repression, which facilitates the design of the process with respect to the use of different sugars. Both enzymes, Tyr1 and Tyr2, were cloned as passengers in this pAIDA1 vector. To reduce proteolytic cleavage of the passenger from the surface^7^ an OmpT-negative strain was employed.

Successful expression of Tyr1 and subsequent melanin formation was detected already from growth on agar plates as the colonies appeared blackish (Fig. 1c). To understand the tyrosinase subcellular localization, deposition was first investigated by Western blotting of soluble (S), inner membrane (IM) and OM cell fractions. The expected 92 kDa band was only present in the OM fraction, confirming positioning of full length Tyr1-AIDA^c^ fusion protein at this location (Fig. 1d). Smaller proteolytic fragments were also detected and in particular, a strong band was detected at approximately 60 kDa, which corresponds to the size of AIDA^c^ without the passenger protein. This result is in accordance with previous findings^16^,^19^,^21^ and demonstrates that inherent proteolytic cleavage cannot yet be fully prevented.

Since the tyrosinase must be exposed to the environment, the orientation in the OM was investigated by flow cytometry. A strong signal from the Myc tag antibody (Fig. 1e), which is positioned closest to the cell surface, indicated that the protein was in the correct orientation i.e. directed away from the outer membrane. The His_6_ tag signal, more exterior from the cell surface, confirms the presence of the full-length protein but as the signal is comparatively weaker this suggests that a proteolytically degraded fraction might be additionally present which is in agreement with the Western blot data (Fig. 1d). To further confirm localization, a protease cocktail was added to cleave surface-displayed enzymes, which resulted in a clear decrease in both fluorescent signals (Supplementary Fig. S1). In contrast to Tyr1, Tyr2 was more severely degraded during translocation to the surface, as the His_6_ tag was almost completely removed whereas the Myc tag was efficiently expressed (Supplementary Fig. S2). We hypothesized that this degradation was likely due to the larger size^16^ of this protein and the presence of cysteine^18^ residues. Homology modeling based on the smaller tyrosinase from *B. megaterium* revealed both C- and N-terminal extensions^23^ (Supplementary Fig. S3). Since these were deemed unnecessary with respect to catalytic activity they were removed to generate a minimized *R. etli* domain (Tyr2_core) of only 36 kDa. Although the His_6_ tag was indeed more efficiently displayed, expression resulted in a broader spectrum of protein variants that decrease the productivity of the full-length protein (Supplementary Fig. S2) and was deemed to be of lesser interest.

**Figure 2:**
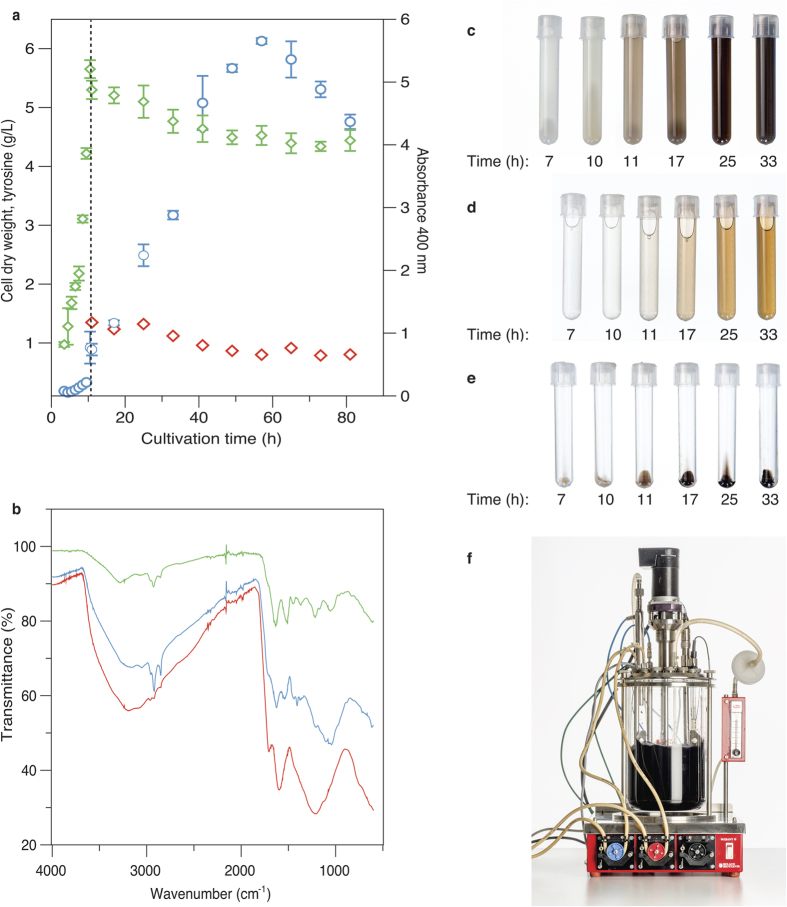
Production of melanin by surface displayed tyrosinase. (**a**) Overview of the batch process for melanin surface expression in the bioreactor. Biomass growth (green squares) and melanin pigment formation (A_400_, blue circles). Melanin pigment formation was observed directly after tyrosine (red squares) addition at 10 hours (dotted line), which occurs concomitant with sugar depletion. (**b**) Fourier transform infrared (FTIR) spectra of synthetic melanin pigments (red), pigments from the cultivation medium (blue) and the cell pellet (green). (**c**–**e**) Time series of pigment formation in the cell suspension (**c**), culture medium without cells (**d**) and cell pellet (**e**). (**f**) Transparent bioreactor showing the cultivation of melanin producing black cells.

Once tyrosinase expression was verified, the biocatalytic reaction was initiated with the addition of tyrosine. Because melanin polymerization from tyrosine is subject to several constraints, different process conditions were screened. This was performed in bioreactors, as constant pH, temperature and oxidative conditions are critical for successful polymerization. Based on these constraints, a batch cultivation was designed in which cells were grown to an optical density (OD_600_) of 2, at which point tyrosinase production was initiated by the addition of the inducer and enzyme cofactor, i.e. copper. Biomass and glucose concentrations were set to allow stable enzyme expression over the course of at least four cell generations before initiation of the melanin reaction. When the carbon source was depleted, tyrosine was added to start melanin synthesis, while dissolved oxygen concentration was maintained above 30% air saturation at all times (Fig. 2a). At the end of the cultivation, a strong dark color had developed in the reactor, and the harvest revealed that the pigment was present both in the cell pellet and in the supernatant (Fig. 2c–f). Repeated washing of the cell pellet led only to a marginal melanin extraction and no visible color shift of the cells, indicating that a large part of the formed melanin remained associated with the cells. This is in contrast to previously reported intracellular tyrosinase production, where the produced melanin has been found in the medium, and not associated to the cell surface^15^,^24^, which has been explained through the excretion of a melanin precursor^15^.

**Figure 3:**
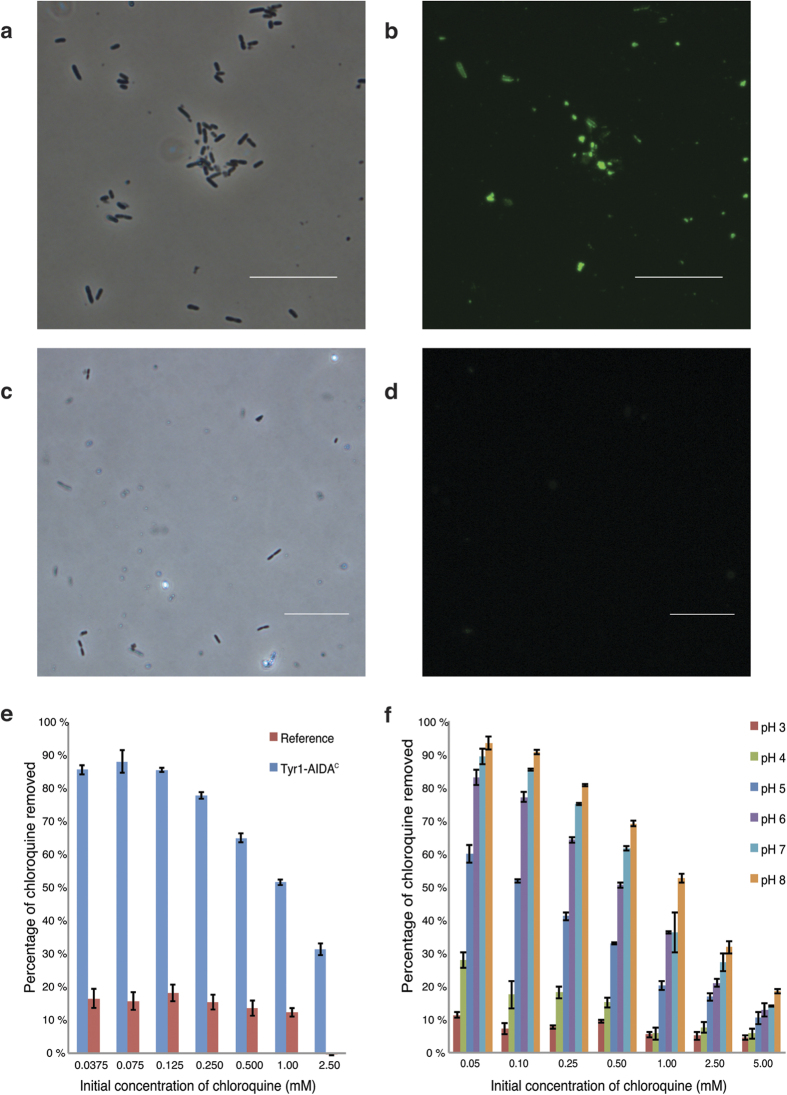
*E. coli* expressing Tyr1-AIDA^c^. Deposition of melanin, absorption and desorption of chloroquine. (**a**–**d**) *E. coli* were probed with fluorescent anti-melanin antibodies and analyzed by fluorescence microscopy. Cells expressing Tyr1-AIDA^c^ were clearly visible (**a**,**b**), whereas reference cells expressing AIDA^c^ without passenger remained unstained (**c**,**d**). White bars in micrographs correspond to 20 μm. Light and fluorescence microscopy images are shown to the left and right, respectively. (**e**) Adsorption efficiency of the antimalarial drug chloroquine to melanin-coated cells. Cells expressing Tyr1-AIDA^c^ (blue) and cells expressing the empty vector (red). (**f**) pH-dependency of cell-chloroquine adsorption. Bar coloring from left: pH 3 (red), pH 4 (green), pH 5 (dark blue), pH 6 (purple), pH 7 (light blue), pH 8 (orange).

To verify that the pigment was indeed melanin, the dark substance was independently extracted from both the cell pellet and the supernatant and compared to a reference of synthetic melanin (Fig. 2b). All compounds exhibited similar Fourier transform infrared spectroscopy (FTIR) spectra, and the general patterns were in agreement with previous melanin data^25^,^26^. As the melanin appeared to remain associated with the cells, we proceeded to investigate the location of the melanin. Because it seemed quite unlikely that the large polymer could pass into the cell interior, we hypothesized that it might instead form a layer coating the cell. To test this hypothesis, pigmented cells were incubated with anti-melanin antibodies and analyzed by fluorescent microscopy. This procedure clearly revealed that melanin was located on the surface (Fig. 3a,b) as the negative control remained unstained (Fig. 3c,d). This difference was also verified with the use of anti-melanin antibodies for flow cytometry analysis (Supplementary Fig. S4). Although false positives might appear from measurements of free melanin particles (particles unattached to the cell surface), the fact that both reference and melanin-expressing cells have similar side- and forward scattering profiles indicates that both sets are equal in size and have similar characteristics and results thus reflect only the cell population (Supplementary Fig. S4a,b).

To test the accessibility and functionality of the melanin surface coated to the cell we thus subjected cells to a specific pharmaceutical. As an example we used chloroquine, an antimalarial drug with a well-established affinity for melanin^9^,^12^ and our results demonstrated that cells coated with melanin successfully adsorbed up to 88% of the chloroquine at concentrations typical for pharmaceutical pollution in wastewater (nanograms to a few milligrams per liter^10^). In contrast, only minor binding was observed to the reference cells (Fig. 3e). Because our results suggest a potential for use in bioremediation we explored further the possibility of regeneration of the system by specific desorption of this pharmaceutical. Different conditions were applied and we found that the binding affinity was largely destabilized by a simple decrease in pH (Fig. 3f), with an approximate reduction of 90% in binding between pH 8 and pH 3. Regeneration could thus be accomplished by a single washing step to retrieve the adsorbed pharmaceutical compound.

In conclusion, we have demonstrated a method to introduce a biocatalytic reaction on the *E. coli* cell surface leading to deposition of the polymer melanin and that this leads to introduction of new cell properties. We showed that these black cells could thus remove a specific drug under conditions resembling those of the outgoing water of a wastewater treatment plant, i.e. where the water is largely depleted of nutrients, particles and general pollution, as it is aimed for release to a recipient, but where pharmaceuticals are still present due to lack of removal technology. In this case, the cells act as passive adsorbents that require no specific conditions for functionality. Furthermore, we suggest a simple regeneration procedure that could contribute to an economic and sustainable process solution. The results of this study are therefore promising for solving one of the most urgent ecological problems today: the accumulation of drug substances in aquatic environments, which is mainly due to household consumption^10^. We believe it likely that this example will promote additional research due to the large binding capacity of melanin for other pollutants^9^,^12^. The applicability of this work is further supported by the generality and multipurpose nature of surface expression and biocatalysis, suggesting the potential use of these technologies in other environmental, industrial and commercial applications.

## Methods

### Strains and plasmids

Table 1 shows a summary of the plasmids and strains used in this study. The plasmid pAIDA1^22^ was used for the surface expression of tyrosinases. This plasmid uses the AIDA-I surface expression system fused to a recombinant passenger protein flanked by an N-terminal His_6_ detection tag and a C-terminal Myc detection tag (Fig. 1a). The strain *E. coli* 0:17ΔOmpT^27^ was used as the host strain for all of the experiments. The tyrosinases used in this study were fused to the autotransporter AIDA-I, generating Tyr1-AIDA^c^ and Tyr2-AIDA^c^. Tyr1 (Supplementary text 1) was synthesized by Eurofins Genomics (Germany) and shows close sequence similarity to the wild type *B. megaterium* tyrosinase^13^. Tyr2 (Genbank accession number: AAM54973) (Supplementary text 2) is identical to the *R. etli* wild type^14^. Both tyrosinases were PCR-amplified from plasmids using the primers (Eurofins Genomics) listed in Table 2 and ligated into the KpnI and SacI restriction sites in pAIDA1. A truncated “core” version of Tyr2 was created using homology modeling with respect to the *B. megaterium* tyrosinase. Based on this homology model, amino acids Ser100-Val412 were amplified using PCR and primers in Table 2 and cloned into pAIDA1, generating Tyr2_core-AIDA^c^ (Supplementary text 2).

### Cultivation media, growth conditions and biomass analysis

Cultivations were performed in a minimal salts medium as previously described^17^ at pH 7.5 and 30 °C in an orbital shaker incubator. The culture medium was supplemented with 100 μg/mL chloramphenicol (Sigma, USA) for plasmid maintenance. For solid medium, a 20X stock of minimal salts was prepared separately and added to 1.5% agar along with all of the aforementioned components. The primary screening of tyrosinase activity was performed using minimal medium agar plates (described above) supplemented with 200 μM isopropyl β-D-1-thiogalactopyranoside (IPTG, VWR, USA), 10 μM CuSO_4_ and 0.5 mg/mL L-tyrosine (Sigma). Bioreactor cultures were performed in a 10-L bioreactor (Belach Bioteknik AB, Sweden) with a working volume of 5 L. The medium was supplemented with 15 g/L glucose, which was sterilized separately and added to the sterile medium in the bioreactor. Cells were grown at 30 °C at a dissolved oxygen tension (DOT) above 30% air saturation. The pH was maintained at 7.5 through automatic titration with NH_4_OH (25% w/v) and H_3_PO_4_ (10% w/v). At an optical density at 600 nm (OD_600_) of 2, tyrosinase production was induced by the addition of IPTG (200 μM) and CuSO_4_ (10 μM). Melanin production was initiated by the addition a of sterile tyrosine stock solution (20 g/L) to a concentration of 1 g/L as the culture entered stationary phase. Fully oxygenated conditions above 30% DOT were maintained. Cell growth was followed by monitoring OD_600_ and measuring cell dry weight (CDW). CDW was measured by collecting 5 mL of cell suspension samples in triplicate into dried, pre-weighed glass tubes. Cells were pelleted by centrifugation (4500 rpm, 5 min), and the resulting cell pellet was dried overnight at 105 °C before weighing. Samples, supernatant and pellets were continuously collected and photographed (Fotograf Lars Nybom AB, Stockholm, Sweden).

### Cell harvest

Cells from bioreactor cultures were harvested by centrifugation (3000 × *g*, 15 min, 4 °C). Cell pellets were resuspended in phosphate buffered saline (PBS), pH 7.0. Both the supernatant and the dissolved cell suspension were stored at 4 °C.

### Biomass and tyrosine quantification

Cell growth was followed by monitoring OD_600_ and measuring cell dry weight (CDW). CDW was measured by collecting 5 mL of cell suspension samples in triplicate into dried, pre-weighed glass tubes. Cells were pelleted by centrifugation (4500 rpm, 5 min), and the resulting cell pellet was dried overnight at 105 °C before weighing. Tyrosine concentrations in the supernatants were analyzed using reversed phase high performance liquid chromatography (HPLC) (Waters) with an AccQTag column (Novapak^TM^ 4 μm C-18 column) and quantified using a UV detector (Waters) at 254 nm. Prior to analysis samples were treated with the AccQTag reagent kit (Waters).

### Flow cytometry

Samples of cells and medium were taken during cultivation and diluted to an OD_600_ of approximately 1, mixed 1:1 with sterile glycerol solution (50% v/v), and frozen at −80 °C for preservation as previously described^17^. On the day of analysis, 50 μL of frozen samples were thawed and washed with 800 μL PBS. After centrifugation (10 min, 4500 rpm, 4 °C), the supernatant was discarded, and the remaining cell pellets were resuspended in 100 μL PBS containing an antibody solution. To detect protein surface expression, 10 μg/mL THE^TM^ His Tag FITC antibodies (Genscript, USA) and 5 μg/mL anti-c-Myc SureLight^®^ allophycocyanin antibodies (Abcam, UK) were used. Samples were incubated at room temperature for 60 min with end-over-end mixing. The solution was then centrifuged (10 min, 4500 rpm, 4 °C) to pellet the cells, washed with 100 μL PBS followed by centrifugation (10 min, 4500 rpm, 4 °C), and resuspended in 500 μL PBS. Surface expression levels were then evaluated by measuring antibody fluorescence in a flow cytometer (Gallios, Beckman Coulter, USA). 10,000 events were recorded per sample. Forward scatter had a discrimination threshold of 7 units, while side scatter contained no discrimination threshold. The same thresholds were used in all experiments.

### Trypsin treatment

The cells were cultivated and induced in shake flasks as described above. Samples were harvested (2000 × *g*, 15 min), redissolved and diluted to OD_600_ = 3 with PBS followed by the addition of 0.5 mg/mL trypsin (porcine pancreas, Sigma). Samples were incubated at 37 °C for 30 min. Trypsin inhibitor (soybean, Sigma) was then added to a concentration of 0.5 mg/mL. Samples were centrifuged (2000 × *g*, 15 min) and washed with PBS.

### Protein isolation from subcellular compartments

Soluble, inner membrane and outer membrane proteins were isolated by the method previously described^28^. Centrifugation (2500 rpm, 15 min, 4 **°**C) was used to harvest 500 mL cultures four generations after induction. Pellets were washed using 140 mL of 50 mM Tris-HCl (pH 7.5) and centrifuged under the conditions described above. 1.75 g of cell pellet was resuspended in 11 mL 50 mM Tris-HCl (pH 7.5) and lysed in a French press high-pressure homogenizer (SLM Aminco, USA). Non-lysed cells were removed by centrifugation (2000 g, 15 min, 4 **°**C). The supernatant was centrifuged (36000 × *g*, 40 minutes, 4 **°**C) to collect the membrane-bound proteins in the pellet. The resulting supernatant containing soluble proteins was collected. The remaining pellet was washed with 1.6 mL 50 mM Tris-HCl (pH 7.5) and centrifuged (36000 × *g*, 40 min, 4 **°**C). The pellet was resuspended in 12 mL 50 mM Tris-HCl with 0.1% (v/v) sarcosyl (*N*-laurylsarcosine sodium salt solution, Fluka, USA) and incubated on a shaking table for 1 hour at 4 **°**C. The suspension was centrifuged (36000 × *g*, 40 min, 4 **°**C), and the supernatant containing the inner membrane was collected. The remaining pellet containing the outer membrane proteins was resuspended in 7 mL 50 mM Tris-HCl with 5 mM EDTA and 1% (v/v) Triton X-100 (SigmaUltra, Sigma). All fractions were stored at −20 **°**C until analysis.

### Western blot

Soluble, inner membrane and outer membrane protein fractions were loaded onto a 10% SDS-PAGE gel (Bis-Tris, NuPAGE, Invitrogen) and run at 180 V. The gel was blotted onto a nitrocellulose membrane and blocked overnight with PBS containing 5% milk powder. The next day, the membrane was incubated for 60 min in PBST containing 10 g/L bovine serum albumin and 0.5 μg/mL antibody (THE^TM^ c-Myc [HRP], GenScript). After washing in PBST, the membranes were developed as previously described^17^,^22^.

### Melanin formation

Formation of melanin was followed by measuring the absorbance at 400 nm (A_400_) of the supernatant from the CDW measurements as previously described^29^. Melanin from 4 mL of supernatant was precipitated by lowering the pH to 2 using HCl followed by centrifugation (4500 rpm, 5 min). To extract cell-associated melanin, the cells were treated overnight with 1 M KOH at 60 °C. Thereafter solutions were filtered (0.2 μm sterile cellulose, VWR), pH was adjusted to 2 and centrifuged for 5 min at 4500 × *g*. The resulting precipitates were washed twice with deionized water, dried overnight at 105 °C and analyzed by FTIR using a Spectrum 2000 FTIR instrument (Perkin–Elmer, USA). The spectra were compared to a reference sample of commercial synthetic melanin (Sigma).

### Chloroquine binding assay

The cells and the drug were dissolved in PBS buffer at pH 7.0 for the initial binding experiments (Fig. 3e). For experiments investigating the adsorption capacities at different pH (Fig. 3f), a 25 mM Na_2_HPO_4_, 25 mM (NH_3_)_2_-H-citrate buffer was used. Cell solutions (5 g/L) and chloroquine solutions were mixed in equal parts to a total volume of 1 mL in microcentrifuge tubes. The samples were incubated (25 °C, 900 rpm) for 30 min in a microcentrifuge tube mixer (Eppendorf, Germany) to avoid sedimentation, followed by centrifugation (16060 g, 10 min). A fraction was pipetted from the top of the vial and analyzed. Chloroquine concentrations varied from 35 μM to 5 mM. All adsorption experiments were performed in triplicate. Chloroquine concentrations in the pH experiments (Fig. 3f) were analyzed using a spectrophotometer (Varian Cary 50, Agilent Technologies, USA) at 343 nm. In other adsorption experiments (Fig. 3e), chloroquine concentrations were analyzed using HPLC (Waters). A Novapak C18 column (Waters) of 3.9 × 300 mm with 4 μm particle size was used. The mobile phase consisted of 15:85 CH_3_CN:Milli-Q supplemented with 1% trimethylamine at a pH of 2.8^30^. Effluent peaks were recorded at 343 nm using a UV detector (Waters), after 3.6 min retention time. The flow rate was 1 ml/min.

### Visualization of melanin by flow cytometry and whole-cell immunofluorescence microscopy

To detect surface-correlated melanin by flow cytometry, the cells were incubated with 6D2 mouse anti-melanin IgM antibody (50 μg/mL) overnight at 4 °C with end-over-end mixing, followed by centrifugation (10 min, 4500 rpm, 4 °C). The pellet was washed with PBS and resuspended in 100 μL PBS with 1.5 μg/mL Alexa Fluor^®^ 488 AffiniPure Donkey Anti-Mouse IgM, (Jackson Immuno, USA) and incubated (1 hour, 25 °C). After incubation, the samples were centrifuged, washed twice, and resuspended in 500 μL PBS for flow cytometry and 100 μL PBS for the visualization of melanin by whole-cell immunofluorescence microscopy. An Olympus BX51 microscope (Olympus, Japan) with a fluorescein filter was used.

## Additional Information

**How to cite this article**: Gustavsson, M. *et al*. Biocatalysis on the surface of* Escherichia coli*: melanin pigmentation of the cell exterior. *Sci. Rep.* **6**, 36117; doi: 10.1038/srep36117 (2016).

**Publisher’s note:** Springer Nature remains neutral with regard to jurisdictional claims in published maps and institutional affiliations.

## Supplementary Material

Supplementary Information

## Figures and Tables

**Table 1:** Strains and plasmids used in this study.

Strains and plasmids	Description	Source
*Strains*		
* E. coli* K12 0:17ΔOmpT	Δ*ompT, sup*^+^, F^−^	23
* E. coli* DH5α	F^−^, *endA1, glnV44, thi-1, recA1, relA1 gyrA96, deoR, nupG, purB20* φ80d*lacZ*ΔM15, Δ(*lacZYA-argF*)U169, hsdR17(*r*_*K*_^−^*m*_*K*_^+^), λ^−^	Invitrogen (USA)
*Plasmids*		
* *pTyr1	Plasmid containing tyrosinase with sequence similarity to *B. megaterium* tyrosinase	This study
* *pAIDA1	AIDA-based surface display vector	3
* *pAIDA1-Tyr1	Fused expression vector containing Tyr1 tyrosinase from pTyr1	This study
* *pTrc99A	Plasmid containing *R. etli* tyrosinase	5
* *pAIDA1-Tyr2_core	Fused expression vector containing *R. etli* core tyrosinase	This study
* *pAIDA1-Tyr2	Fused expression vector containing *R. etli* tyrosinase	This study

**Table 2:** Primers used in this study.

Primer	Sequence
Tyr1_frw_KpnI	AGC GGT ACC GGT AAC AAG TAT AGA GTT AGA AAA AA
Tyr1_rev_SacI	AAA GAG CTC TGA GGA ACG TTT TGA TTT TCT TA
Tyr2_frw_KpnI	AAA GGT ACC GCG TGG CTG GTC GGC AAG
Tyr2_rev_SacI	AGC GAG CTC GGC GGA CAC TAT GGC TAT TTC T
Tyr2_core_frw_KpnI	AAA GGT ACC TCA AAT CCA AGG GAG AGT
Tyr2_core_rev_SacI	AAA GAG CTC GAC CTT GAA GTA GGT CCG

Restriction sites are underlined in the primer sequences.
