# Supplementary material for: Biocatalysis on the surface of Escherichia coli: melanin pigmentation of the cell exterior
Source: Sci Rep. 2016 Oct 26;6:36117. doi: 10.1038/srep36117 (PMC5080590; doi:10.1038/srep36117)
Supplement: Supplementary Information [file srep36117-s1.pdf]

Supplementary information:

Biocatalysis on the surface of *Escherichia coli*: melanin pigmentation of the cell exterior

Martin Gustavsson[1], David Hörnström[1], Susanna Lundh[1], Jaroslav Belotserkovsky[1], Gen Larsson[1]*

[1]Division of Industrial Biotechnology, School of Biotechnology, KTH Royal Institute of Technology, Albanova University Center, SE 10691 Stockholm, SWEDEN

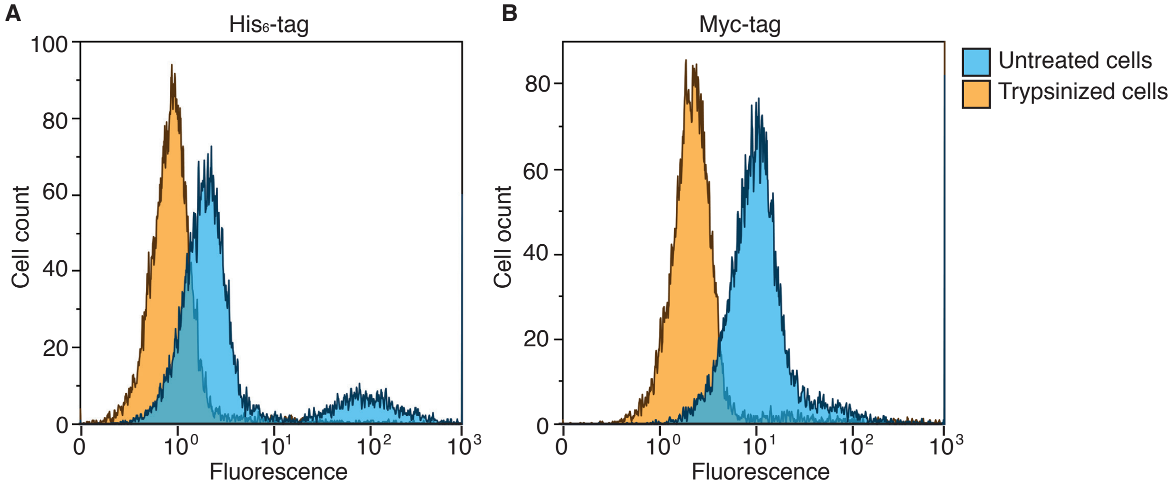

Supplementary fig. S1

Proteolytic treatment of cell surface expressed tyrosinase (Tyr1-AIDA^{c}) in *E. coli* as detected by flow cytometric analysis of fluorescent antibodies against the (**A**) His_6-tag and (**B**) Myc-tag flanking the enzyme. Cells treated with trypsin (yellow) and untreated cells (blue).

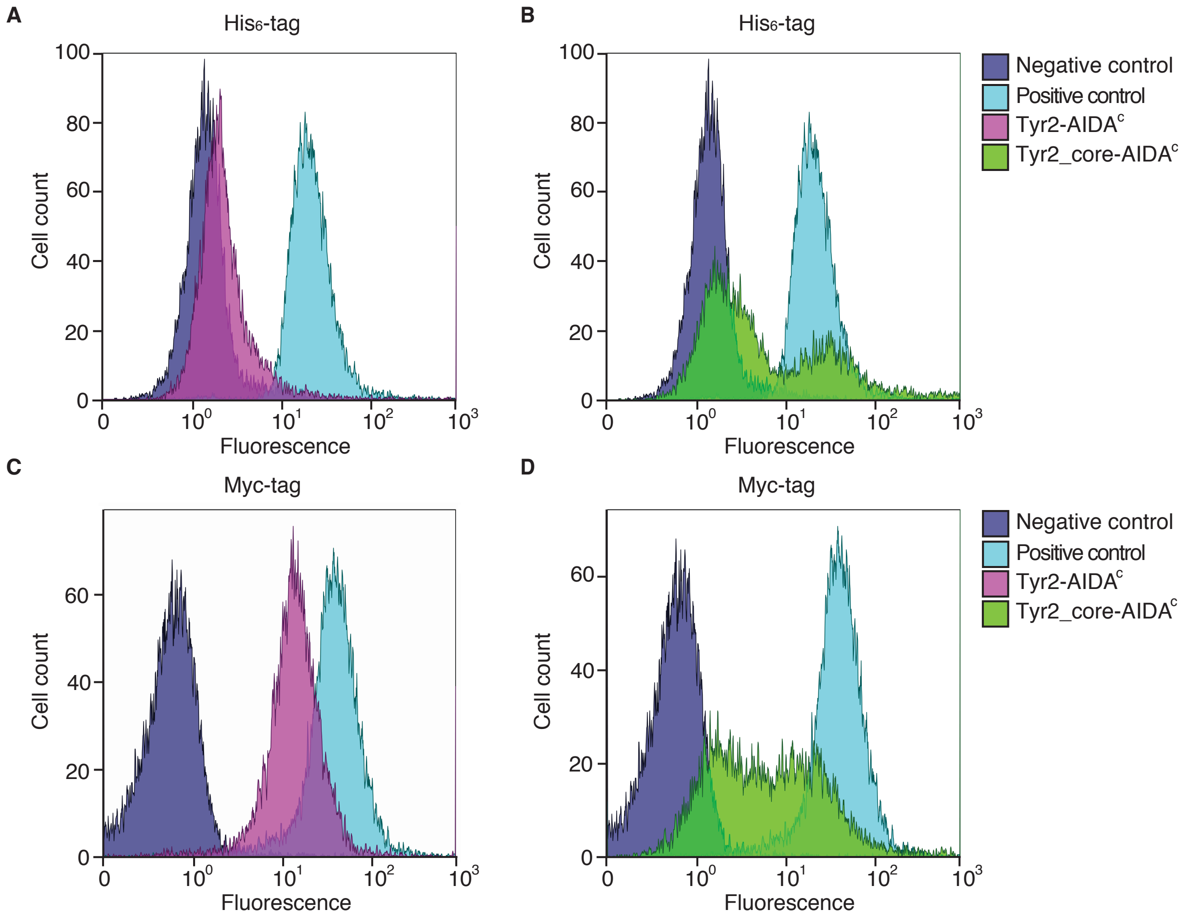

Supplementary fig. S2

Comparison of cells expressing either Tyr2-AIDA^c (**A**, **C**) or the engineered Tyr2_core-AIDA^c enzyme (**B**, **D**) on the surface of *E. coli* cells as detected by flow cytometric analysis of fluorescent antibodies against the His$_6$-tags (**A**, **B**) and the Myc-tags (**C**, **D**). Negative control (**dark blue**) is cells lacking surface expression system, while positive control (**light blue**) is cells expressing the pAIDA1 vector without any passenger.

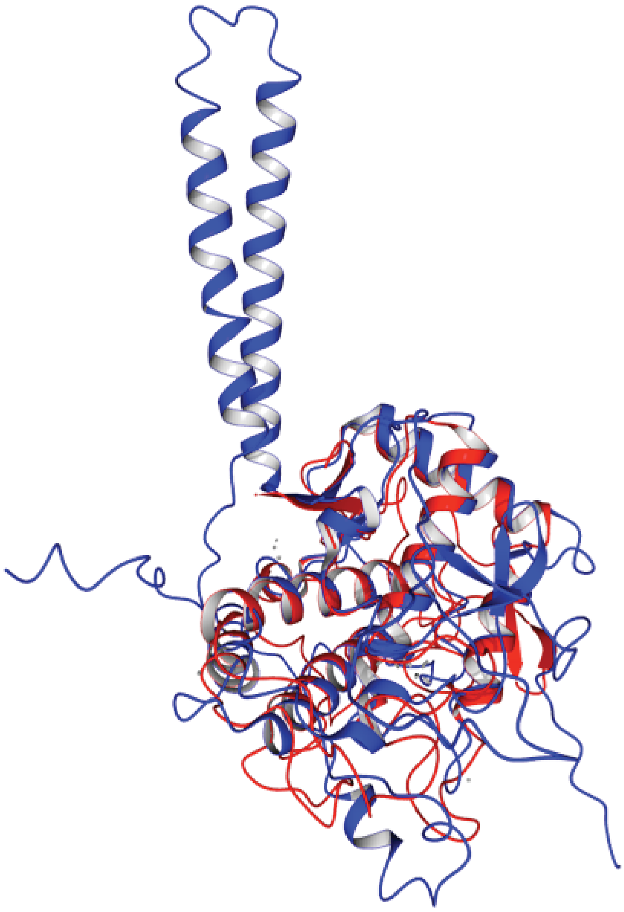

Supplementary fig. S3

Homology model of *R. etli* tyrosinase (**blue**) as positioned at top of the template crystal structure chosen as the *B. megaterium* tyrosinase (**red**) and where the homology model reveals the typical extensions of the C- and N-terminal of this enzyme. The engineered *R. etli-core* tyrosinase (Tyr2_core) was designed by removal of these regions in an attempt to reduce the size and thus increase the possibility for surface display.

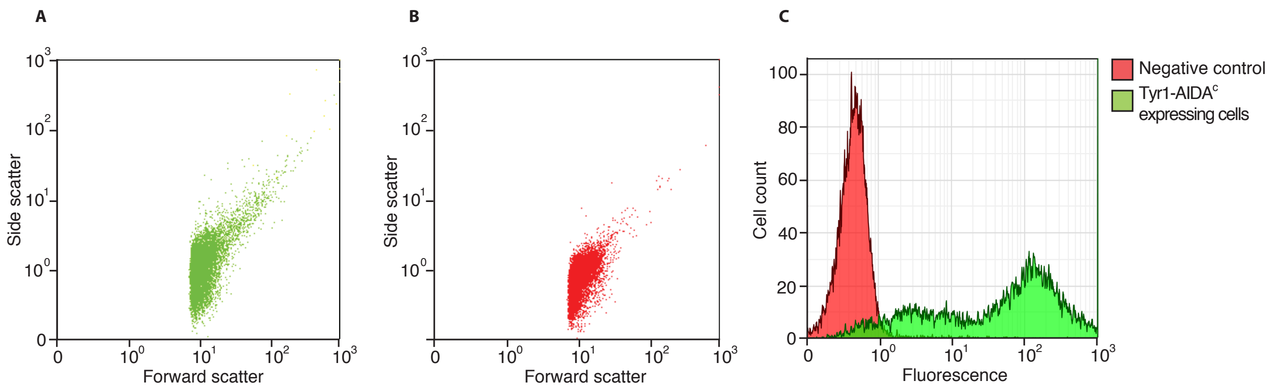

Supplementary fig. S4

Flow cytometric analysis of cells expressing Tyr1-AIDA^c with a melanin coated surface compared to a reference with an empty vector, both treated with 6D2 melanin specific antibodies. Side scatter plotted against forward scatter of cells expressing Tyr1-AIDA^c (**A**), and the corresponding plot for cells expressing the AIDA^c without passenger (**B**). Histogram (**C**) visualizing the fluorescence profile of the Tyr1-AIDA^c expressing cells (**green**) as compared to reference cells expressing AIDA^c without passenger (**red**).

Supplementary text 1

Nucleotide sequence for Tyr1:

ATGGGTAACAAGTATAGAGTTAGAAAAAACGTATTACATCTTACCGAC
ACGGAAAAAAGAGATTTTGTTCGTACCGTGCTAATACTAAAGGAAAAA
GGGATATATGACCGCTATATAGCCTGGCATGGTGCAGCAGGTAAATTT
CATACTCCTCCGGGCAGCGATCGAAATGCAGCACATATGAGTTCTGCT
TTTTTACCGTGGCATCGTGAATACCTTTTACGATTCGAACGTGACCTTC
AGTCAATCAATCCAGAAGTAACCCTTCCTTATTGGGAATGGGAAACGG
ACGCACAGATGCAGGATCCCTCACAATCACAAATTTGGAGTGCAGATT
TTATGGGAGGAAACGGAAATCCCATAAAAGATTTTATCGTCGATACCG
GGCCATTTGCAGCTGGGCGCTGGACGACGATCGATGAACAAGGAAATC
CTTCCGGAGGGCTAAAACGTAATTTTGGAGCAACGAAAGAGGCACCTA
CACTCCCTACTCGAGATGATGTCCTCAATGCTTTAAAAATAACTCAGTA
TGATACGCCGCCTTGGGATATGACCAGCCAAAACAGCTTTCGTAATCA
GCTTGAAGGATTTATTAACGGGCCACAGCTTCACAATCGCGTACACCG
TTGGGTTGGCGGACAGATGGGCGTTGTGCCTACTGCTCCGAATGATCCT
GTCTTCTTTTTACACCACGCAAATGTGGATCGTATTTGGGCTGTATGGC
AAATTATTCATCGTAATCAAAACTATCAGCCGATGAAAAACGGGCCAT
TTGGTCAAAACTTTAGAGATCCGATGTACCCTTGGAATACAACCCCTG
AAGACGTTATGAACCATCGAAAGCTTGGGTACGTATACGATATAGAAT
TAAGAAAATCAAAACGTTCCTCATAA

Supplementary text 2

Amino acid sequence for Tyr2 (Genbank accession number: AAM54973). The underlined amino acids constitute the truncated Tyr2_core.

MPWLVGKPSLERSWNAILSFPESGFQLECRNTIGSSVFSSHFTLHFRVARRLLHFS
CRRFTETQKEPTQALWWCELPTAPAPRRRGTGLKAALILAKDNSNPRESKMSITR
RHVIVQGGVIAAGLLASGLPGTKAFAQIPSIPWRRSLQGLAWNDPIIETYRDAVRL
LNALPASDKFNWVNLSKIHGSGDVVKYCPHGNWYFLPWHRAYTAMYERIVRH
VTKNNDFAMPFWDWTDNPYLPEVFTMQKTPDGKDNPLYVSSRTWPITQPMPDN
IVGPQVLNTILTAKPYEVFGTTRPEGQNSLDPSWVTTSSGTQGALEYTPHNQVHN
NIGGWMPEMSSPRDPIFFMHHCNIDRIWATWNLRNANSTDRLWADMPFTDNFY
DVDGNFWSPKVSDLYVPEELGYNYGFRTYFKVAAASAKTLALNDKLTSVIAAT
ATDAAIAGVTTTSTDNSKAATENVPLSLPIKIPAGALQEIVRQPPLPSGMDTMDFG
AAQEQAASAPRVLAFLRDVEITSASTTSVRVFLGKNDLKADTPVTDPHYVGSFA
VLGHDGDHHRKPSFVLDLTDAIQRVYGGRGQTDGEAIDLQLIPVGSGAGKPGAV
EPAKLEIAIVSA
